# Simulating Macular Degeneration to Investigate Activities of Daily Living: A Systematic Review

**DOI:** 10.3389/fnins.2021.663062

**Published:** 2021-08-13

**Authors:** Anne Macnamara, Celia Chen, Victor R. Schinazi, Dimitrios Saredakis, Tobias Loetscher

**Affiliations:** ^1^Cognitive Ageing and Impairment Neurosciences Laboratory, UniSA Justice & Society, University of South Australia, Adelaide, SA, Australia; ^2^College of Medicine and Public Health, Flinders Medical Centre, Flinders University, Adelaide, SA, Australia; ^3^Department of Psychology, Faculty of Society & Design, Bond University, Gold Coast, QLD, Australia; ^4^Future Health Technologies, Singapore-ETH Centre, Campus for Research Excellence and Technological Enterprise (CREATE), Singapore, Singapore

**Keywords:** age-related macular degeneration, vision impairment, simulation, activities of daily living, rehabilitation

## Abstract

**Purpose:** Investigating difficulties during activities of daily living is a fundamental first step for the development of vision-related intervention and rehabilitation strategies. One way to do this is through visual impairment simulations. The aim of this review is to synthesize and assess the types of simulation methods that have been used to simulate age-related macular degeneration (AMD) in normally sighted participants, during activities of daily living (e.g., reading, cleaning, and cooking).

**Methods:** We conducted a systematic literature search in five databases and a critical analysis of the advantages and disadvantages of various AMD simulation methods (following PRISMA guidelines). The review focuses on the suitability of each method for investigating activities of daily living, an assessment of clinical validation procedures, and an evaluation of the adaptation periods for participants.

**Results:** Nineteen studies met the criteria for inclusion. Contact lenses, computer manipulations, gaze contingent displays, and simulation glasses were the main forms of AMD simulation identified. The use of validation and adaptation procedures were reported in approximately two-thirds and half of studies, respectively.

**Conclusions:** Synthesis of the methodology demonstrated that the choice of simulation has been, and should continue to be, guided by the nature of the study. While simulations may never completely replicate vision loss experienced during AMD, consistency in simulation methodology is critical for generating realistic behavioral responses under vision impairment simulation and limiting the influence of confounding factors. Researchers could also come to a consensus regarding the length and form of adaptation by exploring what is an adequate amount of time and type of training required to acclimatize participants to vision impairment simulations.

## Introduction

Age-related macular degeneration (AMD) is a leading cause of visual impairments, that affects ~200 million people globally (Wong et al., 2014; Jonas et al., 2017), and continues to rise due to the aging population (Velez-Montoya et al., 2014). The vision loss experienced by AMD patients can manifest as a blur, distortion, different colors, or darkness (Taylor et al., 2018a). Vision loss due to non-neovascular AMD can be managed with the support of rehabilitation (Hooper et al., 2008), visual aids (Morrice et al., 2017), or environmental adaptions (Brunnström et al., 2004), but in severe cases of exudative AMD there may be irreversible central vision loss (Jonas et al., 2017). As vision declines, those with AMD report increasing difficulties engaging in activities of daily living (ADL), such as reading, cleaning, and cooking (Bennion et al., 2012; Taylor et al., 2016). Recently, there has been increasing interest into the extent to which AMD affects ADL and quality of life (Jelin et al., 2019; Broadhead et al., 2020; Zult et al., 2020). Characterizing these practical difficulties is an important step in adopting intervention strategies and facilitating positive change for visually impaired individuals.

Previous research has identified difficulties in ADL based upon self-reports from visually impaired patients (Scilley et al., 2002; Walker et al., 2006; Desrosiers et al., 2009). However, directly measuring task performance (e.g., reading, writing, collecting groceries) may be more useful than self-reports because it offers clinicians and researchers an objective assessment of the impact of the visual disability (Culham et al., 2004; Varadaraj et al., 2018; Wittich et al., 2018). Testing visually impaired patients for research purposes can sometimes be challenging because of safety, practical, or availability reasons. In addition, interactive experiments can burden the participants, since visually impaired populations are more likely to have multiple physical and mental comorbidities (Court et al., 2014). Here, it may also be difficult to isolate the effects of visual impairment from the impact of coexisting impairments (e.g., cognitive decline; Wood et al., 2010). One approach to avoid these challenges is to simulate vision loss in normally sighted populations.

Simulation experiments have provided insights into people's behaviors and capabilities with visual impairments (Wood et al., 2010; Lehsing et al., 2019). These experiments have also been used as models for diagnostic visual assessments (de Haan et al., 2020), pilot experiments prior to testing in actual patients (Hwang et al., 2018), and as educational tools for the wider community (Juniat et al., 2019). Critically, simulations can contribute to understanding the effects of eye conditions without subjecting a person to potential risks. For example, Foster et al. (2014) simulated cataracts via goggles on younger adults to examine safety aspects of negotiating stairs on older adults with cataracts. They found that highlighting stair edges with tread increased heel clearance placement and improved safety. Likewise, to assess street-crossing behaviors, participants were positioned on the curb of a street and asked to judge when it would be safe to cross, under normal, and simulated central vision loss conditions (Almutleb and Hassan, 2020). The judgements were similar for both conditions, leading researchers to conclude that eccentric viewing can modulate safety judgements, even with central vision deficits.

Effective simulations of visual conditions can also assist in understanding the effects of eye conditions in order to help develop rehabilitation strategies. Simulation studies have allowed researchers to investigate the manner in which adaptive visual strategies (e.g., pseudofovea) can compensate for vision loss (Barraza-Bernal et al., 2018). Oculomotor adaptations play a fundamental role in visually impaired people learning to use their peripheral vision to reengage with vision-dependent activities (Walsh and Liu, 2014). For example, participants under a central scotoma simulation completed reading tasks after training sessions to induce a preferred retinal locus (Barraza-Bernal et al., 2017). Reading performance significantly improved after each training session, indicating that the task became easier for participants as their ability to use their peripheral vision developed (Barraza-Bernal et al., 2017).

Despite these findings, it still remains unclear what constitutes an “effective simulation.” This could include validation, which refers to the process of determining whether the simulation is an accurate representation of a visual impairment. Valid simulations can be clinically ascertained through mechanisms such as visual acuity or visual field testing. Ensuring that simulations are as realistic as possible is essential when educating others about visual impairments, especially when making practical recommendations.

Effectively constructed simulations may also consider adaptation periods, the time provided to adjust to a simulation before performance is measured. Since AMD is a progressive disease (Taylor et al., 2016; Jonas et al., 2017), patients will typically lose sight over the course of years, allowing them a longer time to adapt to their changing eye condition. This change in vision is in sharp contrast to the immediate loss of vision experienced during a simulation.

Previous research has been critical of simulations of vision loss and blindness. Researchers have suggested that some simulations may be ineffective because they focus on temporary immediate loss of sight (i.e., putting on a blindfold) as opposed to the long-term realities of being blind (Silverman, 2015; Schinazi et al., 2016). Visually impaired patients would likely develop appropriate behavioral and cognitive compensatory strategies over time (Riazi et al., 2013; Rai et al., 2019). In comparison, a normally sighted person's responses under simulation may be exaggerated due to this lack of adjustment. Consequently, a fair and effectively designed simulation study would allow time for normally sighted participants to acclimatize to a simulation (e.g., training sessions, practice trials) prior to measurement (Aguilar and Castet, 2017). While an adaptation period will never replicate the slow progression of vision loss, providing an adequate adaptation period may at least limit confounding behaviors and stressors exhibited as a result of a sudden deprivation of sight. Adaptation to central vision loss has been investigated previously (Kwon et al., 2013; Walsh and Liu, 2014), but to our knowledge, there are no clear guidelines for the suitable length and form of adaptation periods before testing.

The purpose of this paper is to review studies assessing performance in ADL under an AMD simulation in normally sighted people. The importance of investigating specific simulations (e.g., AMD), as opposed to generalized blur simulations, is because the impact on everyday life may vary in response to the condition's predominant manifestation (e.g., AMD affects central vision while glaucoma affects peripheral vision). Synthesizing this literature is intended to provide an overview on the different types of simulation procedures and their suitability for investigating ADL. Investigating the behavioral challenges of ADL (without exposing actual AMD patients to possible psychological and physical harm) is an important step in adopting intervention strategies and facilitating positive change for visually impaired individuals.

## Methods

A systematic search was conducted to identify studies simulating AMD. The review was registered on Open Science Framework (https://osf.io/xkymc). Five electronic databases (Embase Classic + Embase, Ovid Emcare, Ovid MEDLINE® All, Ovid Nursing Database, and PsycINFO) were simultaneously searched via Ovid, on 25th September 2020. A combination of search terms was employed: (“Macular degeneration” OR “Central vision loss” OR “Central scotoma”) AND (“Simulat^*^” OR “Replicat^*^” OR “Imitat^*^” OR “Emulat^*^”). A subsequent updated search was conducted on 30th Match 2021. Preferred Reporting Items for Systematic Reviews and Meta-Analyses (PRISMA) was followed.

Eligible studies were required to be published in English, contain more than five participants (i.e., not case studies), and were original research published in a peer reviewed journal (i.e., no review articles, no conference abstracts). The key criterion was to include studies that simulated AMD within a group of normally sighted people. Studies were excluded if participants had, or have had a history of, any visual impairment, implant, or visual prosthetic. Of primary interest were the methods from the included studies. Descriptions of the AMD simulations (i.e., type and characteristics, validation, and adaptation procedures), the structure of the experiments, and outcome measures were also examined.

Given that symptoms of AMD, specifically central vision loss and central scotomas, can also be indicators of other visual impairments, the screening process was strict in determining the overall purpose of each simulation experiment. As such, articles needed to have cogent reasoning that the purpose of any central vision loss or central scotoma simulation was to primarily mimic AMD, not another condition (e.g., cataracts). However, studies replicating “macular degeneration” were also included because not all researchers use the “age-related” terminology (Copolillo et al., 2017). Articles that were not explicitly related to AMD or macular degeneration were excluded.

Considering our specific interest in AMD's impact on ADL, there were additional restrictions regarding outcome measures. Specifically, studies were excluded if their main focus was on oculomotor behavior (eye movements) and/or vision assessments. Simulation research of this nature is highly informative about fixation and saccade patterns, and the implications of these patterns for the development of a preferred retinal locus or eccentric fixation (Kwon et al., 2013; Costela et al., 2020). However, studies like these do not directly collect data on performance-based measures (e.g., sorting medications) of everyday activities that can immediately inform researchers about the struggles of living with AMD.

Covidence systematic review management software was used to screen the articles (Covidence Systematic Review Software, 2020). Title and abstract screening were conducted by a single reviewer and, followed by two independent reviewers (AM and DS) completing a full text screening. If a consensus could not be reached on a study, a third independent reviewer settled the dispute (TL). Reference lists of the final full texts were screened, via a snowballing strategy, to locate additional studies.

The quality of the included studies was assessed using the Joanna Briggs Critical Appraisal Tools for Quasi-Experimental Studies (Tufanaru et al., 2020). This purpose of these tools are to evaluate the possibility of bias in each study's design, conduct and analysis. Two independent researchers (AM and DS) appraised the methodological quality of the included studies, using a designated checklist of nine criteria. The studies were classed on their risk of bias, depending on the percentage of criteria met (i.e., low, moderate, and high risk = >70%, 50−69%, and <49% criteria, respectively). Cohen's Kappa was calculated to examine the consistency of the independent appraisals.

## Results

**Figure 1:**
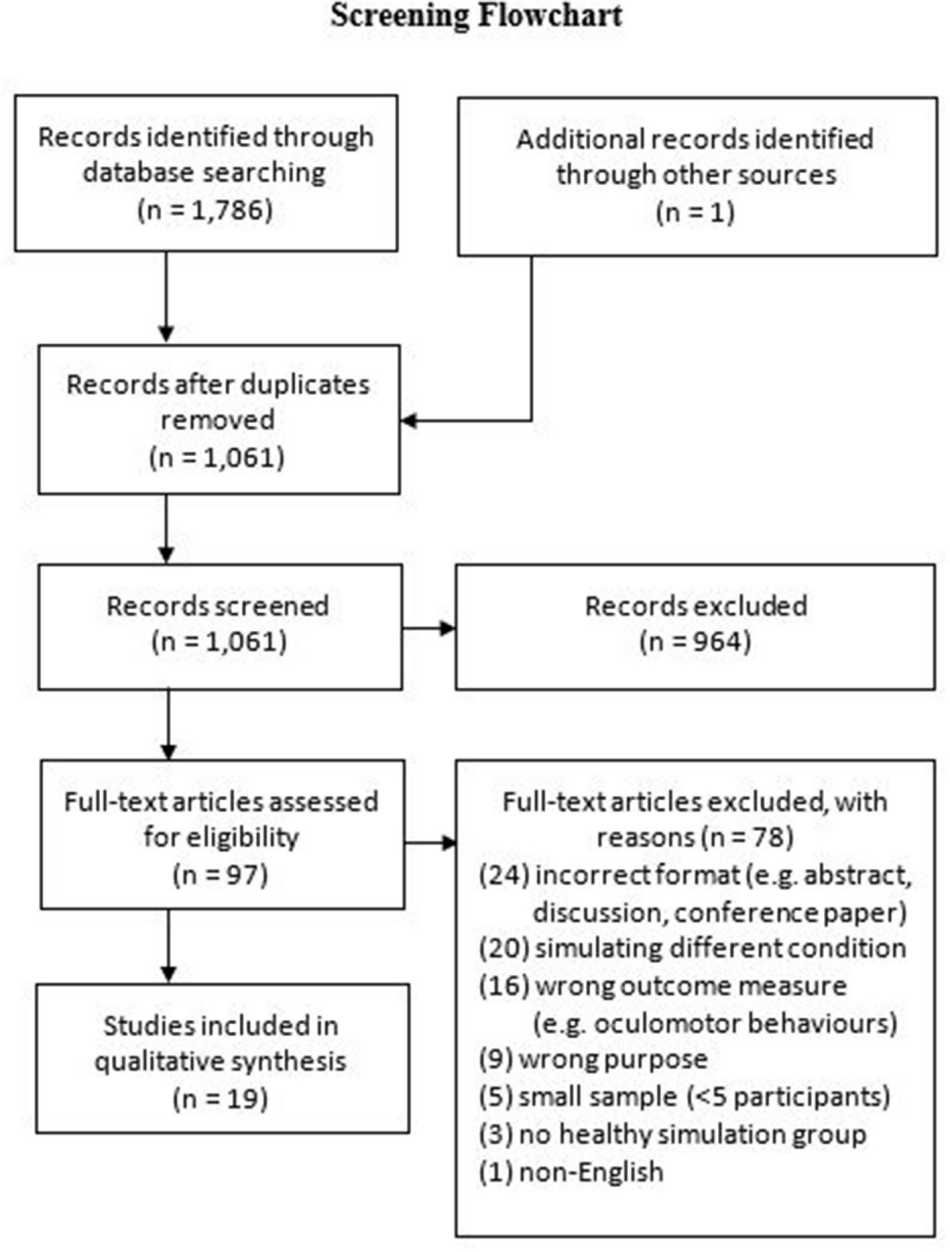
Screening flowchart for studies simulating age-related macular degeneration.

Our database search identified 1,786 publications (see Figure 1 for screening flowchart), with an additional record identified through alternative sources (e.g., reference list searching). Nineteen studies met the inclusion criteria in the final synthesis post-screening. The demographic information, study descriptions, measures, key findings, and risks of bias are presented in Table 1.

**Table 1:** Age-related macular degeneration simulation studies.

**Studies**	**Demographic**			**Study description**	**Tool/Test measure**	**Result/Finding**	**Risk of bias**
	***N***	**Age (M)**	**Sex (F/M)**				
Aguilar and Castet (2017)	10	24.3	5/5	Computer sentence reading task	Comfort rating scale; reading speed	More comfortable using Electronic Vision Enhancement Systems than CCTV (*p* > 0.05) No difference in reading speeds between device groups (*p* <0.05)	Moderate
Almutleb and Hassan (2020)	24	27.0	NS	Street crossing judgement task	Visual acuity (ETDRS and Evans chart); cognitive and health assessments (MMSE, TMT, and SF-36); street crossing judgment scale; street crossing habits scale	No difference in street crossing judgements (accuracy and reliability) between normal and simulated vision (*p* = 0.35 and *p* = 0.09) No correlation between street crossing judgements (accuracy and reliability) and scotoma size (*p* = 0.83 and *p* = 0.95)	Moderate
Bernard et al. (2007)	7	23−43	NS	Computer sentence reading task	Reading speed	Reading speed decreases as scotoma size increases (6°= 84 wpm and 10°= 72 wpm) Reading speed improves as interline spacing increases (6°: *p* = 0.007 and 10°: *p* = 0.004)	Moderate
Copolillo et al. (2017)	10	69	5/5	Walking (indoors and outdoors); stair climbing; carrying laundry and groceries	Center of mass (postural changes); performance speed	More postural changes for activities under simulated vision than normal vision (*p* <0.05 for all except laundry) More stabilization strategies for activities under simulated vision than normal vision (*p* <0.05 for all except sway and therapist assist) Slower performance on activities under simulated vision than normal vision (*p* <0.05)	Low
Czoski-Murray et al. (2009)	105	32	NS	Walking; reading a newspaper, book, and food label; watching TV	Visual acuity (ETDRS and Pelli-Robson chart); health assessment (Health Utilities Index 3, patient time trade-off, and VF-14); performance self-rating scale	Patient time-trade off declined by severity of the vision simulation lens (*p* <0.001) Visual function scores declined as participants unable to perform tasks (*p* <0.001)	High
de Boer et al. (2021)	24	23	15 / 9	Computer emotion identification task	Accuracy	Emotional recognition better when vision intact instead of degraded vision (*p* ≤ 0.01)	Low
Gupta et al. (2018)	13	NS	NS	Computer sentence reading task	Reading speed	Reading speed decreases as scotoma size increases (*p* <0.05) Low vision device remapping increases reading speed around scotomas (exp 1: 4° [*p* > 0.1] and 8° [*p* <0.05]; exp 2: 4° [*p* > 0.1], 8° [*p* > 0.05] and 16° [*p* <0.01]; exp 3: 8°, 12°, and 16° [all *p* <0.01])	Moderate
Ho et al. (2019)	19	18−74	NS	Computer face identity, letter acuity, and sentence reading task	Judgement accuracy; response time; letter acuity (ETDRS and Landolt C); reading speed	Letter acuity improved with reduced pixel size Reading speed slower under simulated vision than normal vision Face recognition slower under simulated vision than normal vision	High
Irons et al. (2014)	31	18−36	19/12	Computer facial dissimilarity and memory task	Judgement accuracy; response time	Facial dissimilarity and discrimination decreased as blur levels increased (*p* = 0.02 and *p* <0.001) Memory performance (accuracy and speed) decreased as blur levels increased (new: *p* <0.001 and *p* = 0.02; old: *p* <0.001 and *p* <0.001)	Moderate
Juniat et al. (2019)	252	NS	NS	Making Tea and filling medicine box	Errors; self-reported difficulty scale	More errors for filling dosette box (0.7) than making tea (0.34) Higher difficulty rating for dosette box (3.23/4) than making tea (2.63/4)	High
Klee et al. (2018)	10	27−43	3/7	Computer letter and pictogram perception tasks	Accuracy	More correct letters (10 and 20°: *p* = 0.01) and pictograms (10° and 20°: *p* = 0.001) with 5 × magnification than 3 × magnification	Moderate
Krishnan et al. (2019)	18	Y: 24−36 O: 55−73	NS	Computer sentence reading task	Reading speed	Reading speed decreases with increasing micro-scotoma density (*p* <0.0001)	Moderate
Kwon et al. (2012)	55	Y: 18−30 O: 55−88	28/27	Computer target identification task	Response time; accuracy; image preference survey	Visual enhancement improved response time for older participants (*p* <0.05 for all except high enhancement) but not younger participants (*p* > 0.05) No difference in accuracy by vision enhancement group (*p* > 0.05) Younger participants responded faster and more accurately than older participants	Moderate
Lane et al. (2019)	74	Y: 20.6 O: 73.3	56/18	Computer emotional expression task	Judgement accuracy	Emotion expression accuracy decreases as blur levels increase (*p* <0.001) Older participants have less accuracy than younger participants (*p* = 0.001) Caricaturing increased expression recognition accuracy (*p* <0.001)	Moderate
McKone et al. (2018)	20	19.0	15/5	Computer facial identity task	Perception rating scale	Caricaturing increased dissimilarity perception (*p* <0.01) Perception improves with increased caricaturing (*p* <0.05)	Low
Rousek and Hallbeck (2011)	50	18−30	25/25	Hospital wayfinding task	Task performance; speed; Wayfinding survey	Wayfinding experience better during normal vision than simulated vision (*p* = 0.005) Faster task completion during normal vision than simulated vision (*p* <0.001)	Moderate
Wensveen et al. (1995)	8	Y: 18−24 O: 62−78	NS	Computer word reading task	Reading speed; accuracy	Reading rates decrease as scotoma size increases (Exp 1: *p* <0.0001 and exp 2: *p* <0.0002) No reading rate differences for older and younger participants (*p* > 0.05) Remapping improves reading rates (*p* <0.01)	Moderate
Wu et al. (2018)	41	18−31	23/18	Street crossing judgement task	Visual acuity (Snellen chart); street crossing judgements and behaviors (gap thresholds, curb delays, crossing times)	Street crossing judgements (gap threshold and curb delay) longer as scotoma size increases (*p* <0.05 for all except 10°/20° scotoma curb delay) No difference in crossing times between groups (*p* > 0.05)	Low
Zagar and Baggarly (2010)	100	20+	66/34	Read pill bottle, patient leaflets, and sort medications	Symptom experience rating scale	Decreased peripheral and central vision, and blurred central vision under simulated vision (*p* <0.0001)	Moderate

*NS, not specified; Y, younger adults; O, older adults; wpm, words per minute*.

*Significance level: p ≤ 0.05*.

A further 16 studies were identified through our search that primarily measured outcomes relating to eye movements, oculomotor behavior, and vision assessments through AMD simulations. However, these studies were beyond the scope our review, which was intended to examine the role of simulation research on investigating ADL performance affected by AMD.

### Critical Appraisal

The reviewers agreed that of the 19 relevant studies, they were generally of moderate methodological quality. The individual assessment of quality originally yielded a moderate level of agreement (*k* = 0.48) prior to discussions and final agreement. The methodological assessment revealed a number of threats to the internal validities for the studies. Also, ~16% of appraisal responses were “unclear.” This occurred when the publications did not report enough to make a sound judgement about the methodology. See Appendix A (Supplementary Material) for the agreed methodological quality assessments for each individual study.

### Age-Related Macular Degeneration Simulation Methods

Methods for simulating AMD can be organized into four main categories (see Table 2) with researchers employing contact lenses, computer manipulations, gaze contingent displays, or simulation glasses.

**Table 2:** Age-related macular degeneration simulation methods.

**Type**	**Studies**	**Apparatus**	**Simulation description**	**Validation procedure**	**Adaptation procedure**
Contact lenses	Almutleb and Hassan (2020)	Soft opaque lens	2.8, 3.0, and 3.2 mm diameter opacity	Tangent screen	Practice task (no time specified)
	Czoski-Murray et al. (2009)	Soft opaque lens	Different sizes black dots	Pilot trials	Practice task (no time specified)
	Klee et al. (2018)	Opaque lens with adjustable adaption device	7.25° dark scotoma	Perimetry	No reported information
Computer manipulation	Irons et al. (2014)	Image manipulation	0°, 10°, 20°, and 30° Gaussian Kernel blur filter	Formula^*^	No reported information
	Krishnan et al. (2019)	Computer generated	2.8°, 5.5°, 8.3°… 16.6° micro-scotomas	Procedure^†^	Practice task (15 min)
	Lane et al. (2019)	Image manipulation	0°, 50°, and 70° Gaussian Kernel blur filter	Formula^*^	No reported information
	McKone et al. (2018)	Image manipulation	0°, 20°, and 30° Gaussian Kernel blur filter	Formula^*^	No reported information
Gaze contingent	Aguilar and Castet (2017)	EyeLink eye tracker	10° gray square scotoma	Calibration (error <1°)	Training session (1 h)
	Bernard et al. (2007)	EyeLink eye tracker	6 and 10° textured and blank square scotoma	Calibration (error <1°)	Practice task (1 h)
	de Boer et al. (2021)	EyeLink eye track	17° × 11.5° semi-circle blurred scotoma	Calibration (error <1°)	Practice trials (no time specified)
	Gupta et al. (2018)^‡^	Tobii TX 300 eye tracker	4°, 8°, and 16° white circle scotoma	Calibration (error <1°)	Practice trials (no time specified)
	Kwon et al. (2012)	EyeLink eye tracker	10° gray circle scotoma	Calibration (error <1°)	Practice trials (no time specified)
	Wensveen et al. (1995)^‡^	SRI dual Purkinje eye tracker	2°, 4°, and 8° gray, opaque circle scotoma	No reported information	No reported information
Glasses	Copolillo et al. (2017)	Goggles	20/400 macular degeneration	No reported information	No reported information
	Gupta et al. (2018)^‡^	Sensics zSight and Oculus DK2 head mounted device/Arrington Research Viewpoint and SMI DK2 Upgrade eye tracker respectively	4°, 8°, and 16° white circle scotoma	Calibration (error <1°)	No practice trials
	Ho et al. (2019)	ODG R-7 augmented reality	20° black opaque tape scotoma	No reported information	Free exploration (few minutes)
	Juniat et al. (2019)	Sim Specs	Age-related macular degeneration	No reported information	No reported information
	Rousek and Hallbeck (2011)	Goggles	20/400 macular degeneration	No reported information	No reported information
	Wu et al. (2018)	NVisor SX60 head mounted device/gaze contingent Arrington eye tracker	10° (20°) and 20° (40°) black circle absolute scotoma (relative blur surrounding scotoma)	Calibration (error <1°)	No reported information
	Zagar and Baggarly (2010)	Goggles	1/2 inch crescent moon black paint; ≤ 20/70 macular degeneration	No reported information	No reported information
Other	Wensveen et al. (1995)^‡^	Material fixed to computer screen	2°, 4°, and 8° gray, opaque, circle film scotoma	No reported information	No reported information

* *See Marmor and Marmor (2010) for previously established formula*.

† *See Krishnan and Bedell (2018) for computer generated procedure*.

‡ *Wensveen et al. (1995) and Gupta et al. (2018) used two methods to simulate age-related macular degeneration*.

#### Contact Lenses

Contact lenses simulate vision loss through varying opacity levels to replicate AMD characteristics (Czoski-Murray et al., 2009; Almutleb and Hassan, 2020). Because contact lenses are placed directly in the eye and capable of following eye movements, researchers can manipulate them to reflect individualized simulation specifications that mimic different stages of AMD.

#### Computer Manipulations

The simulation of AMD via a computer has no direct impact on vision itself. Instead, it presents an end-result representation of what people with visual impairments experience.

#### Gaze Contingent

A gaze contingent simulation is the only method that allows the location of a manufactured scotoma to be continuously realigned in response to a gaze fixation (Aguilar and Castet, 2017; Wu et al., 2018). Due to eye-tracking technology, these simulations can calibrate with a person's pupil. When the person alters their gaze, the simulated scotoma moves to the central region of that new visual field.

#### Glasses

There is great variability in simulation glasses available for visual impairment research. Basic goggles can be self-manufactured to mimic AMD symptoms (e.g., scotoma) using materials such as paint or tape (Zagar and Baggarly, 2010; Ho et al., 2019). Alternatively, simulation glasses can be bought online which are already designed to reflect diminished visual acuity (Connect Solutions Group, 2020). Augmented and virtual reality glasses are more advanced and have the combined benefit of being able to immerse the user into situations that simulate visual impairments while also incorporating gaze-contingent eye-tracking software (Jin et al., 2005; Wu et al., 2018; Jones et al., 2020).

#### Other Methods

Researchers can also develop their own techniques to simulate visual impairment. For example, Wensveen et al. (1995) stuck circular gray film of varying scotoma sizes to a computer screen for participants to read around.

### Validation and Adaptation Procedures

In terms of validation, approximately two-thirds of the studies report information on how the AMD simulations were validated and half of the studies detail procedures how normally sighted participants were adapted to the simulation.

#### Validation

The simulations in 12 studies were authenticated using a variety of clinical and computer modeling techniques (see Table 2). Only two studies attempted clinical validation techniques (e.g., perimetry and tangent screens; Klee et al., 2018; Almutleb and Hassan, 2020). Perimetry and tangent screens are both clinical visual field tests, administered during the diagnosis, and observation of AMD, which can expose the degree of central and peripheral vision loss (Phipps et al., 2004; Acton et al., 2012). The AMD computer manipulations were validated based upon previously developed blurring formulas (Marmor and Marmor, 2010; Krishnan and Bedell, 2018). This includes a computer model that was developed to reformat images relative to the degree of eccentricity (e.g., 5°) and a blur algorithm that simulates symptoms of macular disease created based upon experimental pixilation's of Snellen letters (see Marmor and Marmor, 2010 for more information). Finally, for computer gaze-contingent simulations, studies reported no standardized validation procedures, but rather the removal of trial blocks with calibration errors < 1° (Kwon et al., 2012; Bernard et al., 2016; Aguilar and Castet, 2017; Gupta et al., 2018; Wu et al., 2018; de Boer et al., 2021).

#### Adaptation

As specified in Table 2, nine studies offered their participants training sessions or allowed them to practice the experiment whilst under simulation (Czoski-Murray et al., 2009; Kwon et al., 2012; Bernard et al., 2016; Aguilar and Castet, 2017; Gupta et al., 2018; Krishnan et al., 2019; Almutleb and Hassan, 2020; de Boer et al., 2021). For example, Bernard et al. (2016) provided participants with an hour adaptation session to become accustomed to reading with an artificial scotoma before reading speed was measured. In one study, without training sessions or practice, participants were simply instructed to spend a few minutes looking around their immediate environment whilst wearing simulation glasses (Ho et al., 2019). The remaining studies did not provide additional information to indicate that participants adapted to the simulation prior to testing.

## Discussion

### Comparison of Simulation Methods, Validation, and Adaptation Procedures

There are a series of benefits and drawbacks to each simulation method (summarized in Table 3), which future researchers need to weigh up when designing their own research. For example, in terms of accuracy, gaze contingent simulations may arguably create scotomas that produce the most realistic AMD experience because of the continual realignment of central vision loss in response to changes in eye movements (Aguilar and Castet, 2017). There has been criticism that contact lenses are not capable of emulating the same level of scotoma characteristics (Butt et al., 2015), although newer contact lens designs have attempted to address these limitations (Klee et al., 2018). While still not as precise as gaze contingent paradigms, contact lenses at least retain the advantage of moving together with the eye. In contrast, simulation glasses do not have the level of gaze precision that the former techniques create. Scotomas depicted on most glasses are fixed and will not realign in response to eye movements. Normally sighted participants adopting glasses may simply become accustomed to the simulated characteristics of AMD (e.g., black paint on glasses; Zagar and Baggarly, 2010) and develop strategies (i.e., participants tilting their heads) to look around an artificial blur.

**Table 3:** Advantages and disadvantages of simulation methods.

**Type**	**Advantages**	**Disadvantages**
Contact lenses	Any activity of daily living can be tested (mobility friendly) Individual customization Clinical validation possible	Semi-invasive Infection risk, possible eye irritations Circumventing simulation possible (i.e., seeing past occlusion) Potentially expensive (single use product)
Computer manipulation	Pre-validated (formula based) Limited expense (reusable) Non-invasive	No mobility (limited activities of daily living) Clinical validation not possible Unrealistic visual scanning and compensatory strategies
Gaze contingent	Reasonable accuracy and realism Individual customization Limited expense (reusable) Non-invasive	Limited mobility and activities of daily living (i.e., virtual/augmented reality glasses possible) Clinical validation not possible
Glasses	Any activity of daily living (mobility friendly) Limited expense (depending on product, i.e., homemade) Non-invasive Clinical validation possible	Circumventing simulation possible (i.e., tilting head) Potentially expensive (depending on product, i.e., virtual/augmented reality glasses) Health concerns (i.e., motion sickness)
Other	Limited expense (home-made) Non-invasive	No mobility (limited activities of daily living) Clinical validation not possible

Furthermore, in contrast to gaze contingent paradigms that create unique scotomas for each person, the computer manipulated images are the same for all participants. This approach does not necessarily subvert the quality of computer manipulations given that the descriptions of the methods in each of the papers are based upon standardized formulas reflecting AMD characteristics (Lane et al., 2019). But behavior or performance measured using this method is likely to lack realism. A normally sighted participant assessing a manipulated image will not need to engage in the same compensatory strategies (i.e., using a preferred retinal locus) that people with AMD will do to perform the same task (e.g., judging facial expressions). This limitation is acknowledged by researchers (Irons et al., 2014), who suggest that computer simulations provide practical piloting opportunities prior to testing in patient populations.

Regarding mobility, the gaze-contingent and computer manipulations have previously been limited in the range of ADL that can be investigated. Eye-tracking software used in this research context has typically been computer-based, which means the simulations have naturally given precedence to investigations on stationary behaviors (e.g., reading, facial recognition; Wensveen et al., 1995; Bernard et al., 2016; Aguilar and Castet, 2017; Gupta et al., 2018; Krishnan et al., 2019). Comparatively, contact lenses and glasses are hands free and have allowed the wearer to move around and engage in tasks unimpeded (e.g., walking and carrying laundry; Czoski-Murray et al., 2009; Copolillo et al., 2017). However, with advances in augmented and virtual reality, normally sighted participants can now wear head mounted devices with eye-tracking capabilities (Wu et al., 2018). This means that the benefits of mobility and gaze precision are combined into a single set of simulation glasses. Therefore, future research may evolve to showcase a more diverse range of ADL under gaze-contingent AMD simulations.

Another factor to consider when conducting research is the health and safety of participants. Simulation contact lenses are semi-invasive and participants may not be comfortable inserting them on their eyes. The risks to participants' sight if inserted incorrectly may dissuade participants from taking part in experiments. There are also risks associated with hygiene, as contact lenses can cause corneal infections (Robertson and Cavanagh, 2008). They should not be shared, and therefore could become a costly simulation approach.

The use of augmented and virtual reality simulation glasses may also lead to unwanted side effects (Saredakis et al., 2020). Participants wearing head mounted devices in vision impairment research have reported headaches, nausea, motion sickness (Wu et al., 2018; Deemer et al., 2019; Lorenzini et al., 2019). Such side-effects might result in participants withdrawing from studies or may negatively impact test performance. It may even be challenging to distinguish the outcome effects of the AMD simulation from side-effects of wearing the glasses. Research and technological communities are aware of the limitations of head mounted devices, and efforts to address these issues during the development of newer devices have shown promising results (e.g., less adverse sickness symptomology; Kourtesis et al., 2019). Therefore, the use of augmented and virtual reality glasses as a simulation method in the future may have fewer negative implications on participants' health.

In terms of validation, the use of clinical visual field tests allows the results of a normally sighted person under AMD simulation to be compared with those of a patient with AMD. If the results indicate that a simulation blurs 10° of the visual field, researchers can use this information to conclude whether or not the simulated vision creates an equivalent disruption to central vision as with AMD. However, less direct methods have been used to validate simulations in instances where using clinical diagnostic methods could have been difficult (e.g., the computer manipulations). While the Marmor and Marmor (2010) blur formula to generate AMD images may not be considered a typical validation procedure, adhering to a formula like this is a clever approach for standardizing images that would otherwise be challenging to quantify clinically.

Similarly, gaze-contingent scotomas generated by computers pose barriers for clinical validation. While simulation contact lenses or glasses can be physically worn by participants whilst undertaking a visual field test, computer gaze-contingent simulations cannot. Therefore, researchers rely on the removal of trial blocks with calibration errors (Kwon et al., 2012; Gupta et al., 2018). Since scotomas move with the eye, this technique affords, at a minimum, computer based gaze-contingent simulations a high standard of gaze precision and realism. A visual discrimination task in which foveal processing is tested has been posited to validate gaze-contingent simulations (Geringswald et al., 2013). This method validates simulations based upon the premise that a gaze-contingent scotoma should impair behavioral responses in the same manner that AMD would (Geringswald et al., 2013). More simulation studies should attempt this method in addition to calibration checks.

Virtual visual field tests are another validation process that was not utilized by the studies in this review. Virtual visual field tests can be administered via head mounted glasses, without the assistance of a technician or ophthalmologist, which makes them more accessible than traditional clinical measures. Indeed, there is evidence that the results of virtual visual field tests correlate with that of standard clinical examinations (Wroblewski et al., 2014; Tsapakis et al., 2017). For the AMD simulations, virtual visual field tests could have been administered for contact lens and augmented or virtual reality glasses.

Regardless of whichever validation process is utilized, the importance of validation cannot be underscored. Especially because it is not unusual for researchers to create the simulations themselves. While there might be standard methods employed to manufacture the computer manipulations (i.e., blurring formulas), AMD simulations have also been created by adding paint or tape to glasses (Zagar and Baggarly, 2010; Ho et al., 2019). The consequences of not validating a simulation accurately can have serious real world ramifications. Butt et al. (2015) uncovered such a case when they reassessed a contact lens simulation used by the National Institute for Health and Care Excellence (NICE) for economic evaluations. They found that the contact lenses did not create the reported central scotoma, with the result that the severity of AMD health effects was underestimated with the lenses (Butt et al., 2015). This of real concern if medical professionals and policy makers rely on results derived from poorly validated simulations to justify financial or health care decisions.

Regarding adaptation, it is useful in providing normally sighted participants with the opportunity to adapt to an AMD simulation prior to data collection. Coping strategies, such as relying on different sensory cues or strategically planning alternative behaviors, can be employed by visually impaired people (Riazi et al., 2013; Rai et al., 2019). While those with AMD will have had more time (i.e., months or years) to finesse coping strategies, adaptation periods can still grant normally sighted participants the chance to start mentally strategizing their behavioral adjustments. In experiments of this nature, acknowledging the effect of practice and experience is important in designing a fair comparison. There could be substantial learning effects between the initial moment when participants start a simulation and their performance after a few minutes. Even if the simulation does not exactly replicate vision loss, an adaptation period might be the difference between evaluating an immediate behavior triggered by the reduction in sight and evaluating a more realistic parameter.

Inversely, an adaptation period might also induce unwanted compensatory strategies. For example, during training sessions participants may inadvertently learn ways to circumvent the intended simulation by tilting their heads or squinting (e.g., glasses with opaque lenses). Even when the perceptual deficits of AMD are simulated reasonably realistically and validated, this does not guarantee that normally sighted participants will engage in the same oculomotor behavior as in patients. Participants cannot be compelled to use their peripheral vision rather than evasion techniques. Researchers should prepare for the possibility that normally sighted participants may learn ways to overcome the planned visual deficiencies. Adaptation could therefore be utilized as a method of excluding participants unaffected by vision loss simulations. In one study participants were instructed to centrally fixate on a target while wearing opaque simulation contact lenses, prior to testing (Almutleb and Hassan, 2020). This could have been because people have different resting pupil sizes; therefore, if a participants' pupil was larger than the contact lens occlusion, the participant might have been able to see around the simulated scotoma. In this particular example, four participants were excluded from the study because central fixation was not disrupted as intended (Almutleb and Hassan, 2020). If the researchers had not administered this check, the study may have revealed inaccurate results. The identification of participants seeing pass the simulation is an important reminder that opaque lenses do not always work as intended.

In terms of the length of adaptation periods, researchers have yet to come up with a standard that can be applied in future studies. Studies examining oculomotor strategies have differed in their approaches to inducing a preferred retinal locus in normally sighted participants; training sessions have been conducted over the course of hours, days, or weeks (Kwon et al., 2013; Costela et al., 2020; Maniglia et al., 2020; Prahalad and Coates, 2020). This demonstrates a lack of consistency in how long is considered suitable to reasonable compensate for central vision loss. Within this review, some studies do not specify the length of time while others exposed participants to AMD simulations from a few minutes to up to an hour prior to testing (Aguilar and Castet, 2017; Ho et al., 2019; Krishnan et al., 2019). The few minutes of free visual exploration, offered by Ho et al. (2019), may be preferable to no adaptation time. However, there could be limits to what is learnt regarding practical behavioral changes without specifically guided instructions. Of the other studies which offered adaptation periods, they all included dedicated training or practice with the task. Even though the form of training differed, at least there is a general understanding that training under a simulation is a valuable component of simulation studies. Still, a guideline which offered recommendations on adaptation could help answer questions as to what is the ideal (or minimum) duration and form of training that participants require to acclimatize to vision loss? It is critical to find an answer so that researchers can confidently distinguish that task performance is the direct outcome of any visual simulation. Without adequate adaptation, studies may be confounded by practice effects, as participants initial behavior under simulation may differ from their subsequent behavior by the end of an experiment.

Another consideration to explore is the age of the participants. The average demographic of normally sighted participants in this review is younger adults (see Table 1). This is not reflective of the average age group (i.e., 45–85 years of age) affected by AMD (Jonas et al., 2017). Yet, the recruitment of younger participants is not unusual for all types of simulation studies (Wood et al., 2012; Hwang et al., 2018). In this review, four studies compared younger and older normally sighted adults under simulation (Wensveen et al., 1995; Kwon et al., 2012; Krishnan et al., 2019; Lane et al., 2019). They found significantly decreased performance for both age groups under simulation, but comparatively worse performance for the older adults. A caveat in impairment simulation studies using younger participants may be that the degree to which AMD affects behavior is underestimated. Moreover, in terms of adaptation, if younger participants are performing better than older adults, then it may be possible that they are also adapting to the visual impairment simulations at a faster rate. As such, it is worth exploring whether age affects the required length of adaptation. Researchers could then account for this when interpreting their results in the future and determining adaptation periods.

### What Can We Learn From Simulations?

In this review, many researchers constructed experiments in which task performance was directly compared using normal vision and simulated AMD vision. The studies routinely found significant negative effects under simulation (e.g., slower speed, reduced accuracy) on the respective measured outcomes (see Table 1; Copolillo et al., 2017; Gupta et al., 2018; Lane et al., 2019). For instance, reading focused simulation studies found an expected decrease in reading performance (Wensveen et al., 1995; Gupta et al., 2018; Krishnan et al., 2019), whilst facial recognition studies showed the typical decline in face and emotional perception as simulation of AMD worsens (Irons et al., 2014; McKone et al., 2018; Lane et al., 2019; de Boer et al., 2021). These findings are not surprising as they correspond with similar research findings in AMD populations (Taylor et al., 2018b; Varadaraj et al., 2018). The consistency in the findings implies sufficient AMD simulation accuracy.

Thus, from one viewpoint, it could be concluded that simulation studies are relatively redundant. Instead of offering novel insights into visual impairments, many simulation studies merely confirm findings that can also be established directly from visually impaired participants. However, a unique advantage of AMD simulations is that they allow researchers to control the presentation of symptomology (e.g., size, shape, color, and location of a scotoma). This is not possible when using a clinical population. As AMD is a degenerative eye condition, how ADL are impacted may change as AMD progresses from the non-neovascular to exudative stages. Therefore, the ability to easily alter a scotoma's severity (e.g., 4°, 8°, and 16° scotoma) is a useful manipulation to realistically assess ADL. Significant changes in behavior have been reported in response to different AMD simulation conditions (Wensveen et al., 1995; Krishnan et al., 2019). For example, Wu et al. (2018) found that as the size of a simulated scotoma increased, participants would wait for longer gaps in traffic before deciding to cross a road. This suggests that judging risk is inversely related to the degree of visual decline. An inference such as this can be established quicker by altering an AMD simulation as opposed to recruiting multiple AMD participant groups at different stages of visual decline.

The convenience of control has also been advantageous during investigations into adaptive strategies to loss of central vision (e.g., preferred retinal locus; Barraza-Bernal et al., 2018). This phenomenon may occur if the fovea is damaged during AMD progression (Costela et al., 2020). While not analyzed within this review, simulation research has significantly contributed to our understanding of how a preferred retinal locus can be induced, relocated, or sustained (Kwon et al., 2013; Barraza-Bernal et al., 2017). This knowledge has since been repurposed as a rehabilitative technique to train those with AMD to regain visual ability. Therefore, in the context of studying vision and oculomotor patterns, simulation methods are undoubtably valuable. We acknowledge that by limiting the outcome measures to performance-based measures of ADL, our review may be limited by not examining the benefits that eye movement studies have provided to the visually impaired community.

There is also much that can be learnt from people with AMD on how to improve visual impairment simulations. When patients with AMD were interviewed about their visual loss experience, their descriptions contradicted many widely held beliefs about what AMD looks like (Taylor et al., 2018a). For example, a large proportion of patients reported “missing parts” of their vision, rather than the standard depiction of a central black spot (Taylor et al., 2018a). Many of the studies in this review even portrayed AMD using darkened areas (see Table 2). These portrayals are not inherently false, because some patients do experience black or gray distortions, but it is important to dispel overexaggerated misconceptions. In the future, more researchers could generate AMD simulations by adopting blank scotomas, instead of colored scotomas, to account for patients reporting “missing parts” (Bernard et al., 2007; Macedo et al., 2008).

While clinical validation should be standard practice, feedback from patients can be used to generate and confirm if simulations accurately reflect the deficits they experience (Crabb et al., 2013). One study attempted this by recruiting a group of AMD patients who were visually impaired in just one eye (Denniss and Astle, 2018). The researchers presented images portraying AMD to the participants' unafflicted eye until the depiction of AMD on the picture was an indistinguishable match to that of their afflicted eye (Denniss and Astle, 2018). This method affords researchers an unquestionable representation of AMD that can then be used as a foundation for simulations (although it should still be noted that AMD manifests uniquely for each patient). In general, involving patient populations ensures that from research to clinical care, the perspective and lived experience of being visually impaired is always considered (Dean et al., 2017).

Ensuring authentic simulations is additionally vital when educating the wider community about visual impairments. Simulations have long been utilized in the medical field as a teaching tool to cultivate empathy for patients (Bunn and Terpstra, 2009; Dyer et al., 2018). Simulations allow medical professionals, family members, and the broader community, to metaphorically, “walk in someone else's shoes.” One study found that after completing simple tasks (e.g., making tea) under an AMD simulation, medical students realized how they take their vision for granted and suggested workplace changes to it easier for visually impaired patients (Juniat et al., 2019). This type of self-awareness would be particularly beneficial for family members, who historically become the primary caregiver for their visually impaired family members. Taking care of impaired family members can be a large burden, leading to significant strain on relationships and even depression for the carer (Kuriakose et al., 2017). Ideally, a greater understanding of the difficulties that visually impaired individuals endure may engender more understanding from these individuals to facilitate help with ADL.

In terms of the future of simulation research into ADL's, there is still more that can be explored about the extent to which AMD affects everyday life. At present, there is no comprehensive scale, that can be used by clinicians, that incorporates visual function (e.g., near vision) into how a person performs in their ADL. Of the 19 studies identified in this review, there were more publications on reading alone than physical tasks (e.g., walking, making tea, carrying laundry). This systematic review identifies the need for a unified scale for visual function that incorporates the visual acuity and visual field deficit, as well as a functional scale such as Extended Disability Status Scale (for multiple sclerosis) or Modified Rankins' scale for stroke that incorporate both the static and kinetic tasks to assess independence (Pacific Vision Foundation, 1999). There is no doubt that loss of central vision negatively impacts reading ability (Hamade et al., 2016; Varadaraj et al., 2018), therefore more studies are needed in order to determine the effect of AMD on other daily activities.

There is also potential to broaden the range of outcomes measured. As illustrated in Table 1, studies primarily assessed task performance (e.g., response time, accuracy, errors). Task performance is highly informative of a person's objective ability to complete an activity, but it does not consider the emotional experience of the person whilst completing the task (and the extent to which these emotions affect the completing of the task). Since it is well-established that AMD negatively affects mental health (e.g., anxiety, depression; Williams et al., 1998; Bennion et al., 2012; Cimarolli et al., 2015; Taylor et al., 2016), future studies could examine additional psychological metrics on top of behavioral measures. Although, researchers would need to interpret these findings with caution as the psychological profile of a person experiencing AMD under a short-term simulation may never replicate a patient who lives with AMD every day. Finally, a person with chronic AMD may develop compensatory strategies such as eccentric fixation and preferred retinal locus. As mentioned, these have not been assessed in the 19 studies examined. But repeating ADL performance with AMD simulations may allow further study on these compensatory mechanisms.

## Conclusion

In summary, simulation studies can initiate and complement research into ADL for AMD in a controlled manner. But it is critical to be aware that all simulations have limitations, and none can completely replicate the visual impairments experienced by people living with AMD. As discussed, this is a potential problem when simulations are utilized for economic evaluations. However, for some experimental studies, a simulation that underestimates the true effects of vision loss may not necessarily render the entire simulation useless. For example, if a simulation that underestimates AMD severity can still cause participants to struggle completing an ADL, it suggests that the ADL is likely to be even more difficult for a person with AMD. The use of a specific simulation method will always depend on the nature of the study and ADL under investigation. It can be that some tasks lend themselves to simulation glasses (e.g., wayfinding) whilst others to computer-based gaze-contingent scotomas (e.g., reading). Therefore, the choice of simulation should be considered with the constraints of an experiment task in mind. Accordingly, the validation approach then needs to be suitable for the type of simulation method. A combination of clinical techniques and feedback from AMD patients may be needed to ensure that simulations are as realistic as possible. Regarding adaptation, this field of research would benefit from clear guidelines about what is a reasonable length of time and training type needed to acclimate to vision impairment simulations. Future studies could address this by examining the consistency or progression of task performance after varying adaptation lengths and forms.

## Data Availability Statement

The original contributions presented in the study are included in the article/Supplementary Material, further inquiries can be directed to the corresponding author/s.

## Author Contributions

AM and TL conceptualized the review. AM and DS completed the screening and quality assessment. AM wrote the first draft of the manuscript. AM, CC, VS, DS, and TL contributed to subsequent drafting and the final version of the manuscript. All authors contributed to the article and approved the submitted version.

## Conflict of Interest

The authors declare that the research was conducted in the absence of any commercial or financial relationships that could be construed as a potential conflict of interest.

## Publisher's Note

All claims expressed in this article are solely those of the authors and do not necessarily represent those of their affiliated organizations, or those of the publisher, the editors and the reviewers. Any product that may be evaluated in this article, or claim that may be made by its manufacturer, is not guaranteed or endorsed by the publisher.
