# Supplementary material for: Simulating Macular Degeneration to Investigate Activities of Daily Living: A Systematic Review
Source: Front Neurosci. 2021 Aug 13;15:663062. doi: 10.3389/fnins.2021.663062 (PMC8414246; doi:10.3389/fnins.2021.663062)
Supplement: Supplementary file 1 [file Data_Sheet_1.docx]

Appendix A. The agreed methodological quality assessments, from two reviewers, using the Joanna Briggs Critical Appraisal Tools.

**Checklist criteria:**

1. Is it clear in the study what is the ‘cause’ and what is the ‘effect’ (i.e. there is no confusion about which variable comes first)?

2. Were the participants included in any comparisons similar?

3. Were the participants included in any comparisons receiving similar treatment/care, other than the exposure or intervention of interest?

4. Was there a control group?

5. Were there multiple measurements of the outcome both pre and post the intervention/exposure?

6. Was follow up complete and if not, were differences between groups in terms of their follow up adequately described and analyzed?

7. Were the outcomes of participants included in any comparisons measured in the same way?

8. Were outcomes measured in a reliable way?

9. Was appropriate statistical analysis used?

Joanna Briggs answer options: Y=yes; N=no; U=unclear; NA=not applicable

The studies were classed on their risk of bias, depending on the percentage of ‘Yes’ criteria met: Low risk = >70% Moderate risk = 50-69% High risk = <49%

|  | **Criteria** | | | | | | | | |  |  | **Criteria** | | | | | | | | |  |
| --- | --- | --- | --- | --- | --- | --- | --- | --- | --- | --- | --- | --- | --- | --- | --- | --- | --- | --- | --- | --- | --- |
| **Studies** | **1** | **2** | **3** | **4** | **5** | **6** | **7** | **8** | **9** | **Risk of Bias** | **Studies** | **1** | **2** | **3** | **4** | **5** | **6** | **7** | **8** | **9** | **Risk of Bias** |
| Aguilar and Castet (2017) [A1] | U | Y | Y | N | Y | Y | Y | NA | Y | Moderate | Klee et al. (2018) [A11] | Y | U | Y | N | U | Y | Y | NA | Y | Moderate |
| Almutleb and Hassan (2020) [A2] | U | U | Y | N | Y | Y | Y | NA | Y | Moderate | Krishnan et al. (2019) [A12] | Y | N | Y | N | Y | U | Y | NA | Y | Moderate |
| Bernard et al. (2007) [A3] | Y | N | Y | N | Y | Y | Y | NA | Y | Moderate | Kwon et al. (2012) [A13] | Y | N | U | Y | Y | U | Y | NA | Y | Moderate |
| Copolillo et al. (2017) [A4] | Y | U | Y | N | Y | Y | Y | Y | Y | Low | Lane et al. (2019) [A14] | Y | N | Y | N | Y | U | Y | NA | Y | Moderate |
| Czoski-Murray et al. (2009) [A5] | Y | N | U | N | Y | U | Y | NA | Y | High | McKone et al. (2018) [A15] | Y | Y | Y | N | Y | Y | Y | NA | Y | Low |
| de Boer et al. (2021) [A6] | Y | Y | Y | N | Y | Y | Y | Y | Y | Low | Rousek and Hallbeck (2011) [A16] | Y | Y | Y | N | Y | Y | U | NA | Y | Moderate |
| Gupta et al. (2018) [A7] | Y | U | Y | N | Y | Y | Y | NA | U | Moderate | Wensveen et al. (1995) [A17] | Y | Y | Y | N | Y | N | Y | NA | Y | Moderate |
| Ho et al. (2019) [A8] | U | U | U | N | Y | N | U | NA | U | High | Wu et al. (2018) [A18] | Y | U | Y | Y | Y | Y | Y | NA | Y | Low |
| Irons et al. (2014) [A9] | Y | U | Y | N | Y | U | Y | NA | Y | Moderate | Zagar and Baggarly (2010) [A19] | Y | Y | Y | N | Y | Y | U | NA | U | Moderate |
| Juniat et al. (2019) [A10] | Y | U | U | N | N | Y | Y | NA | U | High |  |  |  |  |  |  |  |  |  |  |  |

**References**

1. Aguilar, C., and Castet, E. (2017). Evaluation of a gaze-controlled vision enhancement system for reading in visually impaired people. *PLoS. One*. 2017;12(4):e0174910. Published 2017 Apr 5. doi:10.1371/journal.pone.0174910
2. Almutleb, E.S., and Hassan, S.E. (2020). The Effect of Simulated Central Field Loss on Street-crossing Decision-Making in Young Adult Pedestrians. *Optom. Vis. Sci*. 2020;97(4):229-238. doi:10.1097/OPX.0000000000001502
3. Bernard, J.B., Scherlen, A.C., and Castet, E. (2007). Page mode reading with simulated scotomas: a modest effect of interline spacing on reading speed. *Vis. Res.* 2007;47(28):3447–3459. doi: 10.1016/j.visres.2007.10.005
4. Copolillo, A., Christopher, A., and Lyons, A. (2017). Effects of Simulated Low Vision on Postural Adjustment to Changes in Center of Mass in Older Adults. *Occup. Ther. Health. Care*. 2017;31(2):115-125. doi:10.1080/07380577.2016.1278295
5. Czoski-Murray, C., Carlton, J., Brazier, J., Young, T., Papo, N.L., and Kang, H.K. (2009). Valuing condition-specific health states using simulation contact lenses. *Value. Health*. 2009;12(5):793-799. doi:10.1111/j.1524-4733.2009.00527.x
6. de Boer, M.J., Jürgens, T., Cornelissen, F.W., and Başkent, D. (2021). Degraded visual and auditory input individually impair audiovisual emotion recognition from speech-like stimuli, but no evidence for an exacerbated effect from combined degradation. *Vis. Res.* 2021;180:51–62. doi.org/10.1016/j.visres.2020.12.002
7. Gupta, A., Mesik, J., Engel, S.A., Smith, R., Schatza, M., Calabrese, A. et al. (2018). Beneficial Effects of Spatial Remapping for Reading With Simulated Central Field Loss. *Invest. Ophthalmol. Vis. Sci*. 2018;59(2):1105-1112. doi:10.1167/iovs.16-21404
8. Ho, E., Boffa, J., and Palanker, D. (2019). Performance of complex visual tasks using simulated prosthetic vision via augmented-reality glasses. *J. Vis*. 2019;19(13):22. doi:10.1167/19.13.22
9. Irons, J., McKone, E., Dumbleton, R., Barnes, N., He, X., Provis, J. et al. (2014). A new theoretical approach to improving face recognition in disorders of central vision: face caricaturing. *J. Vis*. 2014;14(2):12. Published 2014 Feb 17. doi:10.1167/14.2.12
10. Juniat, V., Bourkiza, R., Das, A., Das-Bhaumik, R., Founti, P., Yeo, C. et al. (2019). Understanding Visual Impairment and Its Impact on Patients: A Simulation-Based Training in Undergraduate Medical Education. *J. Med. Educ. Curric Dev*. 2019;6:1-7. Published 2019 May 9. doi:10.1177/2382120519843854
11. Klee, S., Link, D., Sinzinger, S., and Haueisen, J. (2018). Scotoma Simulation in Healthy Subjects. *Optom. Vis. Sci*. 2018;95(12):1120-1128. doi:10.1097/OPX.0000000000001310
12. Krishnan, A.K., Queener, H.M., Stevenson, S.B., Benoit, J.S., and Bedell, H.E. (2019). Impact of simulated micro-scotomas on reading performance in central and peripheral retina. *Exp. Eye Res.* 2019;183:9–19. doi: 10.1016/j.exer.2018.06.027
13. Kwon, M., Ramachandra, C., Satgunam, P., Mel, B.W., Peli, E., and Tjan, B.S. (2012). Contour enhancement benefits older adults with simulated central field loss. *Optom. Vis. Sci*. 2012;89(9):1374-1384. doi:10.1097/OPX.0b013e3182678e52
14. Lane, J., Robbins, R.A., Rohan, E.M.F., Crookes, K., Essex, R.W., Maddess, T. et al. (2019). Caricaturing can improve facial expression recognition in low-resolution images and age-related macular degeneration. *J. Vis*. 2019;19(6):18. doi:10.1167/19.6.18
15. McKone, E., Robbins, R.A., He, X., and Barnes, N. (2018). Caricaturing faces to improve identity recognition in low vision simulations: How effective is current-generation automatic assignment of landmark points?. *PLoS. One*. 2018;13(10):e0204361. Published 2018 Oct 4. doi:10.1371/journal.pone.0204361
16. Rousek, J.B., and Hallbeck, M.S. (2011). The use of simulated visual impairment to identify hospital design elements that contribute to wayfinding difficulties. *Int. J. Ind. Ergon.,* 2011;41(5):447-458. doi:10.1016/j.ergon.2011.05.002
17. Wensveen, J.M., Bedell, H.E., and Loshin, D.S. (1995). Reading rates with artificial central scotomata with and without spatial remapping of print. *Optom. Vis. Sci*. 1995;72(2):100-114. doi:10.1097/00006324-199502000-00009
18. Wu, H., Ashmead, D.H., Adams, H., and Bodenheimer, B. (2018). Using Virtual Reality to Assess the Street Crossing Behavior of Pedestrians With Simulated Macular Degeneration at a Roundabout. *Front. ICT.,* 2018;5;:27. doi:10.3389/fict.2018.00027
19. Zagar, M., and Baggarly, S. (2010). Low vision simulator goggles in pharmacy education. *Am. J. Pharm. Educ*. 2010;74(5):83. doi:10.5688/aj740583
